# Chemical Composition of the Essential Oils of the Flowers, Leaves and Stems of Two *Senecio polyanthemoides* Sch. Bip. Samples from South Africa

**DOI:** 10.3390/molecules14062077

**Published:** 2009-06-09

**Authors:** Lawal A. Oladipupo, Oyedeji O. Adebola

**Affiliations:** Department of Chemistry, University of Zululand, KwaDlangezwa 3886, South Africa

**Keywords:** *Senecio polyanthemoides* Sch. Bip., asteraceae, essential oil composition

## Abstract

The essential oils of the flowers, leaves and stems of *Senecio polyanthemoides* Sch. Bip. Samples collected from two different localities within the city of uMhlathuze, KwaZulu-Natal Province (South Africa) were isolated by hydrodistillation and analyzed using GC and GC/MS. Twenty-six constituents were identified, representing an average of 86.0 - 99.6% of the total oil composition. The chemical profile reveals the dominance of monoterpenoid compounds, although some quantitative variance was noticed. The main constituents of the oils were limonene (3.1 – 43.0%), *p*-cymene (4.9-36.3%), *β*-selinene (1.3-32.7%), *α*-pinene (1.8-21.4%), *β*-pinene (7.6-16.5%) and 1,8-cineole (9.3-11.4%), caryophyllene oxide (4.1-13.4%) and humulene epoxide II (8.6-10.3%).

## Introduction

The genus *Senecio* (family *Asteraceae*; tribe *Senecioneae*) consists of more than 1,500 species of aromatic herbs and shrubby plants native to Southern Europe, but now spread all over the world. A few herbaceous species of the genus are grown as ornamental plants [1,2]. Literature reports on the phytochemistry of these species shows a large variety of pyrrolizidine alkaloids [3] and sesquiterpenoids [4], diterpenoids [5], triterpenoids [6], shikimic acid and cacalolide derivatives [7,8]. Furthermore, Biological activities such as antibacterial [9], molluscicidal [10], antimicrobial [11] and cytotoxic activities [12], and biosynthesis of algal pheromones [13] have been reported for these plants. In traditional medicine, the use of *Senecio* species for wound healing and treatment of coughs, bronchitis, asthma and eczema have been reported [14,15]. In the flora of South Africa, there are about 300 species of the genus *Senecio* with over 120 species found in KwaZulu-Natal Province [16,17]. The leaves are alternate in arrangement and the flowers are variously coloured (mostly yellow, but blue, purple or white forms are also found). Many species of the genus *Senecio* have been reportedly used by the Sotho, Xhosa and the Zulu tribes of South Africa as traditional remedies for colds and sore throats, coughs, burns and wounds, enemas in chest complaints, nausea and vomiting, stomach ache, hiccups, purgatives and also for anal protrusion in children, blood purifiers for skin eruptions and treatment of venereal diseases [18,19,20].

*Senecio polyanthemoides* Sch. Bip. is a bushy annual herb of about 1.8 m height with a woody stem at the base. The leaves (15 x 4 cm) are lanceolate, petiole-like and pinnately divided with smooth and thinly white-felted upper and lower surfaces respectively. Inflorescences are radiate, with many short bracteates arranged in spreading corymbose panicles and flowers mainly in September and October. *S. polyanthemoides* grows naturally on forests margins, farmlands and in scattered localities along roadsides in KwaZulu-Natal [16,17]. 

**Figure 1:**
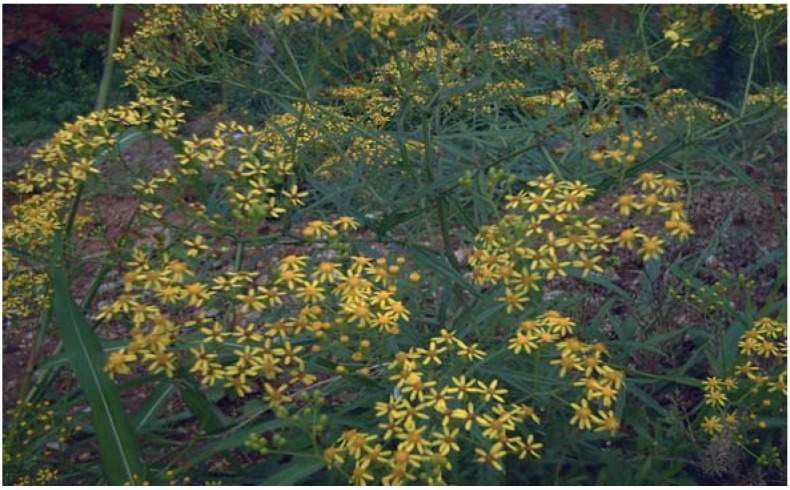
*Senecio polyanthemoides* plants.

The chemical composition of the essential oils of some *Senecio* species have been reported [21,22,23,24,25,26,27,28,29,30,31,32]. The volatile oils from the aerial parts of *S. nutans* Sch. Bip collected from two different localities in Arequipa and in different seasons of the year from Luara region, both in Peru, Southern America, showed that monoterpene hydrocarbons predominated in all the oils. In the oils of Arequipa, sabinene and *α*-terpinene were the main constituents; while, the oils of the Luara region had *α*-phellandrene and *p*-cymene as the principal components. The leaf oil of *S. squalidus* L. from France was found to contain *p*-cymene (29.3%) and *α*-phellandrene (24.7%) as the major components. The essential oil of *S. farfarifolius* Boiss. Et Kotschy from Turkey was report to contain *α*-pinene (48.3%) and 1.8-cineole (10.3%) as the predominant constituents of the oil. The volatile constituents of *S. glaucus* subsp*. coronopifloius* from Belgium have myrcene (24.0%) and dehydrofukinone (21.0%) as the major components. The Indian species was found to be a potential source of *α*-thujone (84.17%). Furthermore, two Iranian *Senecio* species, *S. leucostachys* Baker essential oil has sabinene (20.7%), *α*-phellandrene (19.7%), germacrene D (10.8%) and *β*-caryophyllene (8.2%) ; while, *S. vernalis* Waldst. & Kit. had spathulenol (37.1%), 1,8-cineole (19.0%), *m*-cymene (16.6%), isobicyclogermacrenal (15.2%) and *α*-phellandrene (3.4%) as their major compounds. Also, the essential oils of *S. aegyptius* var*. discoideus* Boiss from Egypt have 1,10-epoxyfuranoeremophilane as the main component of the oils [9]. To the best of our knowledge, there are no reports on the essential oil profile of *Senecio* species growing in South Africa. Therefore, this paper reports for the first time the chemical composition of essential oils of flowers, leaves and stems of two *Senecio polyanthemoides* collected from two different locations in KwaZulu-Natal, South Africa.

## Results and Discussion

Table 1 lists the physical properties of the essential oils of *S*. *polyanthemoides*. As shown in the table, hydrodistillation of the flower, leaf and stem samples of *S*. *polyanthemoides* from two different locations gives pale yellow oils in yields of 0.10 – 0.23% for Sample A and 0.07 – 0.21% for Sample B. The percentage composition of the essential oils and the fragmentation pattern of the components identified are listed in order of their elution off a DB-5 column (Table 2).

**Table 1:** Physical properties of essential oils of *Senecio polyanthemoides* from two different locations.

	*Yield (%)*	*Color*	*Odour*	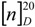
Sample A				
Flower	0.10	Pale yellow	aromatic	1.4726
Leaf	0.23	Pale yellow	spicy	1.4731
Stem	0.17	Pale yellow	herbaceous	1.4720
Sample B				
Flower	0.07	Pale yellow	aromatic	1.4347
Leaf	0.21	Pale yellow	spicy	1.4320
Stem	0.11	Pale yellow	herbaceous	1.4320

In sample A (Table 2), 13 constituents (97.0%) were identified in flower oil: nine monoterpenes (97.0%) and three sesquiterpenes (traces). *p*-Cymene (24.7%), limonene (18.3%) and myrcene (15.7%) were the major components. Eight constituents (94.1%) were identified in leaf oil: six monoterpenes (48.0%) and two sesquiterpenes (51.6%). *β*-Selinene (32.7%) was the most abundant constituent in this oil, followed by caryophyllene oxide (13.4%), *α*-pinene (11.8%) and 1,8-cineole (11.4%). In the stem oil, eight constituents (99.6%) were identified, with six being monoterpenes (85.3%) and two sesquiterpenes (14.3%). This oil was characterized by the presence of *α*-pinene (21.4%), *p*-cymene (18.7%), limonene (18.1%), *β*-pinene (12.4%) and 1,8-cineole (9.3%).

In sample B (Table 2), 21 constituents (94.3%) were identified in flower oil: 13 monoterpenes (92.0%) and eight sesquiterpenes (2.3%). *p*-Cymene (36.3%) was again the most abundant constituent; others were *β*-pinene (16.5%), myrcene (14.3%) and *α*-phellandrene (9.5%). The leaf oil has 12 identified constituents (99.5%): eight monoterpenes (91.0%) and four sesquiterpenes (8.5%). Myrcene (31.7%), limonene (19.7%) and *Z*-(*β*)-ocimene (15.9%) were the major components. In the stem oil, seven constituents (86.0%) were identified: Five monoterpenes (71.6%) and two sesquiterpenes (14.4%).

**Table 2:** Chemical composition of essential oil of *Senecio polyanthemoides* Sch. Bip.

(%) Composition								
		Sample A			Sample B			
Compounds	RRI							m/z
		F	L	S	F	L	S	
*α*-pinene	938	7.4	11.8	21.4	1.8	7.0	4.2	93, 79, 41,136
sabinene	972	3.5	3.2	5.4	tr	4.0	-	93, 77, 41, 136
*β*-pinene	981	7.6	7.6	12.4	16.5	7.8	tr	93, 41, 79, 136
myrcene	991	15.7	-	-	14.3	31.7	4.3	41, 93, 69, 136
*α*-phellandrene	1003	2.7	-	-	7.8	-	-	93, 77, 41, 136
*p*-cymene	1022	24.7	5.3	18.7	36.3	4.9	20.1	119, 91, 77, 134
limonene	1029	18.3	8.7	18.1	3.1	19.7	43.0	68, 93, 79, 136
1,8-Cineole	1031	-	11.4	9.3	-	-	-	43, 81, 55, 154
*Z*-(*β*)-ocimene	1027	4.4	-	-	3.8	15.9	-	93, 79, 41, 136
*E*-(*β*)-ocimene	1035	8.3	-	-	7.1	tr	-	91, 79, 41, 136
*γ*-terpinene	1056	-	-	-	0.3	-	-	93, 77, 121, 136
terpinolene	1083	-	-	-	0.3	-	-	93, 121, 79, 136
linalool	1096	-	-	-	0.6	-	-	43, 71, 55, 154
terpinen-4-ol	1176	4.4	-	-	0.1	-	-	71, 41, 93, 154
*α*-copaene	1373	-	-	-	0.1	-	-	119, 161, 105, 204
*β*-caryophyllene	1421	-	-	-	0.6	-	-	41, 91, 79, 204
*α*-humulene	1452	-	-	-	0.9	2.9	-	93, 80, 41 204
(*Z,E*)-*α*-farnesene	1457	-	-	-	0.1	-	-	93, 41, 135, 204
germacrene D	1479	tr	-	-	-	0.3	2.4	161, 105, 91, 204
*β*-selinene	1482	-	32.7	-	-	1.3	-	107, 93, 121, 204
unknown	1487	tr	-	-	-	1.9	-	41, 121, 93, 204
(*E,E*)-α-farnesene	1494	-	-	-	0.1	-	-	41, 93, 69, 204
*δ* -cadinene	1518	-	-	-	0.1	-	-	161, 119, 134, 204
caryophyllene oxide	1572	tr	13.4	5.7	-	-	4.1	41, 79, 93, 220
humulene epoxide II	1596	-	-	8.6	-	-	10.3	43, 67, 109, 220
farnesol	1719	-	-	-	0.1	-	-	41, 69, 81, 93
								
Monoterpene hydrocarbons		92.6	36.6	85.3	92.0	91.0	71.6	
Oxygenated monoterpenes		4.4	11.4	9.3	0.1	-	-	
Sesquiterpene hydrocarbons		-	32.7	-	2.3	8.5	-	
Oxygenated sesquiterpenes		-	13.4	14.3	0.1	-	14.4	
Total identified		97.0	94.1	99.6	95.3	99.5	86.0	

RRI = Retention relative index to C_9_-C_24_ n-alkanes on DB-5 column; tr. = trace amount (< 0.05%); F = flowers; L = leaves; S = stems; Sample A = Owen Sithole College of Agriculture, Empangeni; Sample B = KwaDlangezwa road, off University of Zululand, KwaDlangezwa.

Analyses of the oils shows that they were predominantly monoterpenoid in nature, like some other species in the genus *Senecio*. The oils were characterized by large amount of monoterpenes (48.0 – 97.0%), except for the leaf oil of sample A with a higher amount of sesquiterpenes (46.1%).

**Figure 2:**
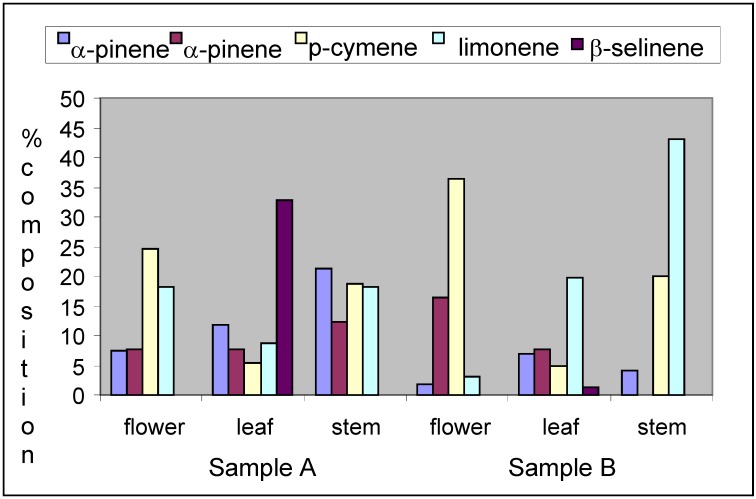
Major components of the flower, leaf and stem oils of *Senecio polyanthemoides* from two different locations.

In terms of the organs studied, that is the flower, leaves and stem, the flowers of sample B had β-pinene (16.5%) α-phellandrene (7.8%) and p-cymene (36.3%) in susbtantial quantity, compared to sample A with β-pinene (7.6%) α-phellandrene (2.7%) and p-cymene (24.7%). Other compounds identified in sample B such as linalool, γ-terpinene, α-humulene, β-caryophyllene were completely absent in the flower oil of sample A. The flower oil of sample A on the other hand could be a good source of limonene, considering the compositional concentration (18.3%) when compared to 3.1% in sample B. This effect was noticeable in the odour of the flowers as that of sample A was stronger than that of sample B. The leaves of sample B had myrcene (31.7%), α-humulene (2.9%) and germacrene D (2.4%) as major or significant compounds present in the oil and not found in the Sample A leaf oil. In contrast, sample A had β-selinene (32.7%) and 1,8-cineole (11.4%) as major components. Variation in the compositional concentration of some compounds was also observed in the two leaf oils. The concentration of limonene (19.7%) in sample B was double the amount identified in the leaf oil of sample A (8.7%). Noteworthy is the concentration of limonene also in the stem oil of sample B which accounts for the major component of the oil. Considering the compositional variation, it could be said that the leaves and stem sample A would be of more medicinal value due to the presence of 1,8-cineole when compared to sample B, that did not have this chemical compound present in the oils. However, the stems of Sample B can be a very good source for limonene, which suggests its uses in flavoring applications. Figure 2 shows the major components of the oils of *Senecio polyanthemoides* from two different locations. In our own opinion, the two samples are completely different thus leading us to suspect that they are likely to be different chemotypes.

Comparing the present data (Table 1) with those previously reported in literature, the studied essential oils displayed different chemical profiles, although monoterpene hydrocarbons have been reported as the main constituents of the essential oils of several species of the genus *Senecio* [25,27,28,30,31], which are similar to the South Africa species. However, some species of this genus are characterized by high percentage of oxygenated compounds and mainly furanoeremophilan, as was found in *Senecio chrysanthemoides*, *Senecio vernalis* and *Senecio aegyptius* var. *discoideus* [22,32,9], and were not detected in the present study. Interestingly, limonene, which was found to be a major component in our study, has not been reported as the main constituent in any *Senecio* species. Many reports have shown how plant growth and development are affected by genetic and environmental factors, and how these factors contributes to differences in chemical variation of essential oils of plants with different chemotypes [33,34,35,36,37,38,39]. The chemical variation of essential oils of different chemotypes of *Thymus* species from different locations or growing in the same habitat have been attributed to different in environmental and genetic factors [33,34,35,36,37]. Furthermore, ecological factors, particularly, light and temperature have also been reported to influences the production of essential oils as well as other active agents in plants [38,39].

## Experimental

### Plant Material

*Senecio polyanthemoides* plants growing wild on the campus of Owen Sithole College of Agriculture, Empangeni and along KwaDlangezwa road, opposite University of Zululand, KwaDlangezwa, in the city of uMhlathuze, KwaZulu-Natal Province, South Africa, at a distance of about 120 Km from each other were randomly collected at flowering stage in September, 2006. The taxonomic identification of the plant materials was confirmed by a senior plant taxonomist, Dr S.J. Siebert of the Department of Botany, University of Zululand, KwaDlangezwa. Voucher specimens [Lawal, OA 23 & 24 (ZULU)] were deposited at the University of Zululand, Herbarium.

### Oil isolation

Fresh flowers (115 g), leaves (300 g) and stems (500 g) of each sample were separately subjected to hydrodistillation in a Clevenger-type apparatus for 3h in accordance with the British Pharmacopoeia specification [40,41,42]. Briefly, sample was added to 750-1.5 mL of distilled, deionized water in a 2-5 L round-bottomed flask and heated to boiling, after which the essential oil was evaporated together with water vapour and finally collected in a condenser. The upper phase that contained the essential oil was separated from the lower one and the distillate isolated was preserved in a sealed sample tube and stored under refrigeration until analysis. 

### GC analyses

GC analyses was carried out on a Hewlett Packard HP 6820 Gas Chromatograph equipped with a FID detector and HP-5 MS column (60 m x 0.25 mm id, 0.25 µm film thickness) and split ratio of 1:25. Column temperature was initially kept at 50 ^o^C for 2 min, then gradually increased to 240 ^o^C at a 5 ^o^C/min rate, and was held for 10 min. Injection and detector temperatures were 200 ^o^C and 240 ^o^C respectively. Hydrogen was the carrier gas, at a flow rate of 1 mL/min. A 1.0 µL aliquot of the diluted oil was injected into the GC. Peaks were measured by electronic integration. *n*-Alkanes were run at the same condition for Kováts indices determination.

### GC-MS analyses

GC-MS analyses of the oils were performed using a Hewlett Packard Gas Chromatography HP 6890 equipped with a HP-5 MS capillary column (30 m x 0.25 mm id, film thickness 0.25 µm) interfaced with a Hewlett Packard 5973 mass spectrometer system. The oven temperature was programmed from 70 - 240 ^o^C at the rate of 5 ^o^C /min. The ion source was set at 240 ^o^C and electron ionization at 70eV. Helium was used as the carrier gas at a flow rate of 1 mL/min, with a 1:25 split ratio. Scanning range was 35 to 425 amu. A sample (1.0 µL) of diluted oil in hexane was manually injected into the GC/MS.

### Identification of compounds

The components of the oils were identified base on the comparison of their retention indices and mass spectra with those standards, Wiley Library mass spectra database of the GC/MS system and published data [43,44,45].
